# Stochastic Entropy Production Associated with Quantum Measurement in a Framework of Markovian Quantum State Diffusion

**DOI:** 10.3390/e26121024

**Published:** 2024-11-26

**Authors:** Claudia L. Clarke, Ian J. Ford

**Affiliations:** Department of Physics and Astronomy and London Centre for Nanotechnology, University College London, Gower Street, London WC1E 6BT, UK

**Keywords:** stochastic entropy production, quantum measurement

## Abstract

The reduced density matrix that characterises the state of an open quantum system is a projection from the full density matrix of the quantum system and its environment, and there are many full density matrices consistent with a given reduced version. Without a specification of relevant details of the environment, the time evolution of a reduced density matrix is therefore typically unpredictable, even if the dynamics of the full density matrix are deterministic. With this in mind, we investigate a two-level open quantum system using a framework of quantum state diffusion. We consider the pseudorandom evolution of its reduced density matrix when subjected to an environment-driven process that performs a continuous quantum measurement of a system observable, invoking dynamics that asymptotically send the system to one of the relevant eigenstates. The unpredictability is characterised by a stochastic entropy production, the average of which corresponds to an increase in the subjective uncertainty of the quantum state adopted by the system and environment, given the underspecified dynamics. This differs from a change in von Neumann entropy, and can continue indefinitely as the system is guided towards an eigenstate. As one would expect, the simultaneous measurement of two non-commuting observables within the same framework does not send the system to an eigenstate. Instead, the probability density function describing the reduced density matrix of the system becomes stationary over a continuum of pure states, a situation characterised by zero further stochastic entropy production. Transitions between such stationary states, brought about by changes in the relative strengths of the two measurement processes, give rise to finite positive mean stochastic entropy production. The framework investigated can offer useful perspectives on both the dynamics and irreversible thermodynamics of measurement in quantum systems.

## 1. Introduction

In classical statistical mechanics, entropy quantifies uncertainty in the adopted configuration of a system when only partial detail is available concerning the coordinates of the component particles. This is a subjective uncertainty, a reflection of the personal state of ignorance of a given observer. The capacity of an observer to predict future behaviour when such a system is coupled to a similarly underspecified environment is limited and their knowledge of the state worsens with time, even if the dynamics are entirely deterministic. The total entropy of the system and environment increases as a consequence. In many situations, such evolution can be associated with the dissipation of potential energy into heat, and this underpins the role played by entropy in the (19th century) second law of thermodynamics [1,2,3].

The 21st century concept of entropy production, however, is based on mechanics, specifically a consideration of the probabilities of forward and backward sequences of events governed by an effective stochastic dynamics. In this framework of ‘stochastic thermodynamics’, entropy change is the expectation value of a ‘stochastic entropy production’, clarifying a number of long-standing conceptual issues [4,5,6,7,8].

**Figure 1:**
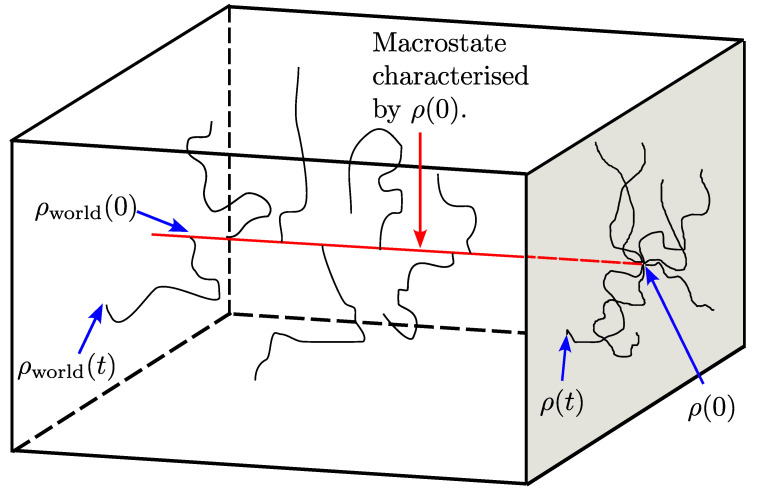
The box and the grey area represent the phase spaces of the density matrix of the world $\rho_{\mathrm{world}}$ and of the reduced density matrix ρ of a constituent open system, respectively. Deterministic trajectories $\rho_{\mathrm{world}}\left(t\right)$ that start at t=0 from a macrostate subspace (shown as a red line), characterised by a given initial value ρ(0) of the reduced density matrix, can be manifested as pseudorandom trajectories for ρ(t) in the reduced phase space, shown as projections onto the right-hand face.

The central aim of this paper is to employ entropy as a description of uncertainty of the adopted configuration at the level of a reduced density matrix in quantum mechanics. Putting aside the issue of quantum measurement for the moment, the full density matrix of a system together with its environment (a closed ‘world’) evolves deterministically according to the unitary dynamics of the von Neumann equation. This can give rise to an evolution of the reduced density matrix describing the system that preserves unit trace and positivity but allows changes to von Neumann entropy and purity, corresponding to thermalisation, for example [9,10,11]. But the trajectory followed will be unpredictable if the complete initial state of the world is not specified. It is natural to regard this as producing an effective Brownian motion of the reduced density matrix, and to concern ourselves with the associated entropy increase. The concept is illustrated in Figure 1. This intrinsic unpredictability holds whether or not we introduce ideas of randomness associated with quantum mechanical measurement.

In developing this idea, we view the reduced density matrix as an analogue of classical system coordinates and hence as a physical description of the quantum state, not merely as a vehicle for specifying probabilities of projective measurements or a representation of a state of knowledge. But coordinates that describe the physical state of a system ought not to change discontinuously, which would seem to raise difficulties in connection with the instantaneous projections normally considered to arise from quantum measurement. If the density matrix represents a physical state, we are therefore obliged to model quantum measurement in a fashion that avoids discontinuous jumps.

To this end, we pursue the idea that quantum measurement, namely the adoption by a system of an eigenstate of an observable when interrogated by a measuring device, is brought about by the deterministic dynamics of the density matrix describing the system and its environment, of which the measuring device would be a part. We explore the idea that quantum measurement arises from the unitary dynamics of the world, its apparent stochasticity being a consequence of a failure to specify the initial degrees of freedom of the environment, or more precisely those of a measuring device. Such an origin of stochasticity is reminiscent of ideas employed in classical statistical mechanics.

In such a scheme, the evolution of the system under the influence of its environment would be governed by a nonlinear dynamics with attractors corresponding to the appropriate eigenstates. It is not our aim here to derive such nonlinear dynamics from an underlying evolution of the world. Instead, we seek a model of system dynamics that has the desired effect, namely that the reduced density matrix under measurement should evolve along continuous trajectories, terminating at eigenstates.

The modelling of ‘weak measurement’ in quantum mechanics produces continuous stochastic quantum trajectories [12,13,14]. Random incremental changes in the state of an open system are brought about by projective measurements of remote parts of the environment. We shall employ this mathematical framework (without the associated narrative of remote projective measurement) to represent the envisaged nonlinear dynamical interactions between the system and environment that guide the system towards eigenstates of observables under certain conditions [15].

The framework known as quantum state diffusion (QSD) [16,17,18,19,20] is a broad category of open quantum system dynamics that can represent the phenomenology of weak measurement. A continuous, Markovian, stochastic evolution of the reduced density matrix emerges. More elaborate schemes are also possible, for example, involving non-Markovian dynamics. The approach can be used to model the continuous measurement of an open system that is consistent with strong projective measurements as a limiting behaviour and is compatible with the Born rule. Measurement in QSD is a process driven by specific system–environment coupling and takes place without discontinuities [21,22,23]. This is a quantum dynamics that resembles classical dynamics, but where the dynamical variables are the elements of a reduced density matrix. It combines both aspects of quantum evolution: determinism of the von Neumann equation together with stochasticity representing measurement or more general environmental effects [24].

The idea that quantum jumps are not instantaneous but merely very rapid is not an unusual one [25] and the non-locality of quantum mechanical evolution remains intrinsic to the interpretation. Nevertheless, such a viewpoint is not without its controversies [26,27,28,29]. In particular, a suggestion that the quantum state represents a physical configuration of the world might appear to conflict with various positions taken in the fundamental interpretation of quantum mechanics, for example, those where a physical state (‘reality’) is considered to be induced by the projective measurement process. Moreover, the supposed ‘hidden variables’ carried by the system and the environment, ignorance of which gives rise here to the effective stochastic evolution, might seem to conflict with the breakage of Bell inequalities and other similar statistical results [30,31]. Resolution of this issue might involve a deeper consideration of the implications of determinism [32]. Alternatively, one could simply regard quantum state diffusion as merely a mathematical framework for modelling continuous pseudorandom quantum evolution.

If the evolution of the reduced density matrix can be modelled in a fashion that avoids discontinuities, then the concept of stochastic entropy production in quantum mechanics can be introduced in a straightforward way [33,34,35,36,37,38,39,40,41,42]. Entropy production arising from evolution that includes quantum jumps can also be considered, but this introduces difficulties that manifest as infinities in the change in system Gibbs entropy [37]. We believe that such problems ought to be avoided if possible.

When dynamical variables evolve according to Markovian stochastic differential equations (SDEs), or Itô processes [43], it is possible to derive a related Itô process for the stochastic entropy production [7]. This allows us to compute a stochastic entropy production associated with individual Brownian trajectories taken by the reduced density matrix of a system. For situations where the system is guided towards an eigenstate of an observable, we can compute the stochastic entropy production characterising a process of measurement.

A positive expectation value of such a stochastic entropy production represents increased subjective uncertainty in the quantum state of the world. Growth in uncertainty is natural since we model the evolution using stochastic methods starting from an incompletely specified initial state. The state of the system can become *less* uncertain, a necessary aspect of the performance of measurement, but uncertainty with regard to the state of the rest of the world will increase by a greater amount, thereby allowing the second law of thermodynamics to be satisfied. It should be noted that stochastic entropy production here does not correspond to a change in von Neumann entropy, which instead describes the uncertainty of outcome when a system is subjected to projective measurement in a specific basis. We comment further on this in Section 2.5.

In Section 2, we develop these ideas in the context of the measurement of a single observable in a two-level quantum system starting in a mixed state [44]. Mean stochastic entropy production is found to be positive and without limit as the system is guided, asymptotically in time, into one or the other of the two eigenstates. We go on in Section 3 to consider the simultaneous measurement of two non-commuting observables and show how the stochastic entropy production is finite, a consequence of the inability of the dynamics, in this situation, to guide the system into a definite eigenstate of either observable.

We interpret the results in Section 4 and summarise our conclusions in Section 5, suggesting that dynamics based on quantum state diffusion, with an interpretation of the reduced density matrix as a set of physical properties of a state, together with the use of stochastic entropy production to monitor the process of eigenstate selection, can provide some conceptual clarification of the quantum measurement problem [31].

## 2. Measurement of $\sigma_{z}$

### 2.1. Dynamics

We consider a two-level system described by a reduced density matrix (hereafter, simply a density matrix ρ) defined in a basis of eigenstates |±1〉 of the $\sigma_{z}$ operator. Pure states denoting occupation of one of the two levels correspond to $\rho_{\pm }^{e}=\left|\pm 1\rangle \langle \pm 1\right|$. Starting in the mixed state $\rho =\frac{1}{2}\left(a_{+}\rho_{+}^{e}+a_{-}\rho_{-}^{e}\right)$, where $a_{\pm }$ are real coefficients, we use a quantum state diffusion approach to model the stochastic evolution of the system into one or the other of the levels in accordance with the Born rule.

We consider a minimal scheme [13] employing a rule for stochastic transitions given by

(1) $$\rho \to S^{\pm }\left(\rho \right)={\rho \prime }^{\pm }=\frac{M_{\pm }\rho M_{\pm }^{†}}{\mathrm{Tr}\left(M_{\pm }\rho M_{\pm }^{†}\right)},$$

with

(2) $$M_{\pm }=\frac{1}{\sqrt{2}}\left(I-\frac{1}{2}c^{†}cdt\pm c\sqrt{dt}\right),$$

where $c=\alpha_{z}\sigma_{z}$, with real scalar parameter $\alpha_{z}$ designated as the strength of measurement. The $M_{\pm }$ are examples of *Kraus operators*, and the map in Equation (1) often appears in descriptions of physical transformations of a density matrix. The probabilities for the selection of one of the two possible outcomes ${\rho \prime }^{\pm }$ after an infinitesimal timestep dt are

(3) $$p_{\pm }\left(\rho \right)=\mathrm{Tr}\left(M_{\pm }\rho M_{\pm }^{†}\right)=\frac{1}{2}\left(1\pm C\sqrt{dt}\right),$$

where $C=\mathrm{Tr}\left(\rho \left(c+c^{†}\right)\right)$. The quantum map in Equation (1) preserves the trace of ρ. Furthermore, since the Kraus operators in Equation (2) differ incrementally from (a multiple of) the identity, the positive definiteness of ρ is maintained [24]. The operator identity $M_{+}^{†}M_{+}+M_{-}^{†}M_{-}=I$ is also satisfied. This scheme defines a stochastic dynamics representing the effect of a device interrogating the occupation of levels of the system, whereby the eigenstates of $\sigma_{z}$ are stationary, i.e., $p_{+}\left(\rho_{+}^{e}\right)=p_{-}\left(\rho_{-}^{e}\right)=1$, $p_{-}\left(\rho_{+}^{e}\right)=p_{+}\left(\rho_{-}^{e}\right)=0$, and $S^{+}\left(\rho_{+}^{e}\right)=\rho_{+}^{e}$, $S^{-}\left(\rho_{-}^{e}\right)=\rho_{-}^{e}$.

The two possible increments $d\rho^{\pm }={\rho \prime }^{\pm }-\rho$ available in the timestep dt under the dynamics are

(4) $$\begin{array}{cc} d\rho^{\pm } & =\left(c\rho c^{†}-\frac{1}{2}\rho c^{†}c-\frac{1}{2}c^{†}c\rho \right)dt-\left(\rho c^{†}+c\rho -C\rho \right)Cdt\\ \; & \;\pm \left(\rho c^{†}+c\rho -C\rho \right)\sqrt{dt}, \end{array}$$

and by evaluating the mean and variance of this increment in ρ, it may be shown that the evolution can also be represented by the Itô process

(5) $$d\rho =\left(c\rho c^{†}-\frac{1}{2}\rho c^{†}c-\frac{1}{2}c^{†}c\rho \right)dt+\left(\rho c^{†}+c\rho -C\rho \right)dW,$$

where dW is a Wiener increment with mean 〈dW〉=0 and variance $\langle dW^{2}\rangle =dt$, with the brackets representing an average over the stochasticity. Note that terms of higher order than linear in dt will be neglected throughout. A continuous evolution of the stochastic variable ρ driven by the infinitesimal stochastic variable dW has emerged, analogous to a random walk or Brownian motion. This is what is meant by quantum state diffusion.

A process of averaging then leads to the standard Lindblad equation [45]:

(6) $$\frac{d\bar{\rho }}{dt}=c\bar{\rho }c^{†}-\frac{1}{2}\bar{\rho }c^{†}c-\frac{1}{2}c^{†}c\bar{\rho },$$

with $\bar{\rho }=\langle \rho \rangle$. Such a deterministic equation describes the average dynamical behaviour of an ensemble of density matrices. The actual trajectory followed by a system as it responds to external interactions, however, is specified by the stochastic Lindblad Equation (5) [46,47]. The environment disturbs the system in a manner represented by one of the transformations or moves given in Equation (1), selected at random with probabilities (3) that arise from the underspecification of the environmental state and hence of $\rho_{\mathrm{world}}$ in Figure 1.

If we represent the density matrix in the form $\rho =\frac{1}{2}\left(I+r_{z}\sigma_{z}\right)$, it may be shown that the dynamics of Equation (5) correspond to the evolution of the real stochastic variable $r_{z}\left(t\right)$ according to [13]

(7) $$dr_{z}=2\alpha_{z}\left(1-r_{z}^{2}\right)dW.$$

**Figure 2:**
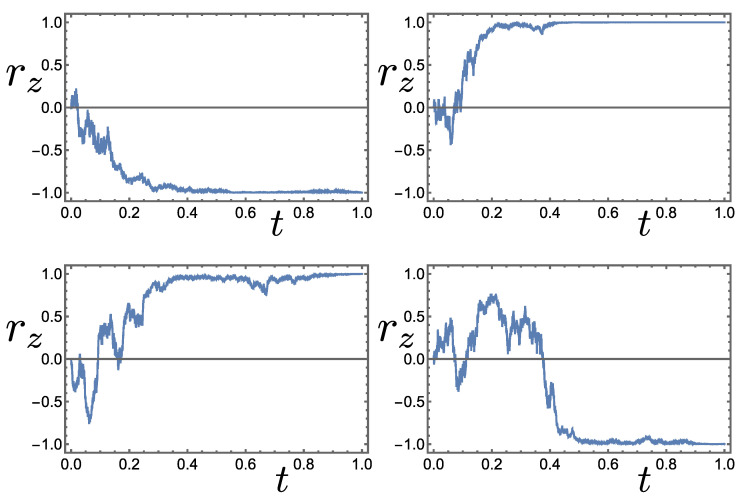
Four stochastic trajectories $r_{z}\left(t\right)$ derived from Equation (7) with strength of measurement $\alpha_{z}=1$. Starting at $r_{z}\left(0\right)=0$, they evolve towards eigenstates of the $\sigma_{z}$ observable at $r_{z}=\pm 1$.

Example realisations of such behaviour, starting from the fully mixed state at $r_{z}\left(0\right)=0$, are shown in Figure 2. Notice that $r_{z}$ evolves asymptotically towards ±1, corresponding to density matrices $\rho_{\pm }^{e}$, and note also that the average increment $\langle dr_{z}\rangle$ over the ensemble satisfies $\langle dr_{z}\rangle =d\langle r_{z}\rangle =2\alpha_{z}\left(1-\langle r_{z}^{2}\rangle \right)\langle dW\rangle =0$, implying that $\langle r_{z}\rangle$ is time-independent and that 〈ρ〉 is as well. A similar conclusion can be reached simply by evaluating the right-hand side of Equation (6).

The standard Lindblad equation cannot capture system ‘collapse’ to an eigenstate, but instead describes the average behaviour of an ensemble of collapsing systems. For a closer consideration of the dynamics and thermodynamics of collapse, we need to ‘unravel’ the standard Lindblad equation into its stochastic version (5), using it to generate an ensemble of trajectories that model possible physical evolutions of the open quantum system.

Using Itô’s lemma, it can be shown that the purity of the state, $P=\mathrm{Tr}\rho^{2}=\frac{1}{2}\left(1+r_{z}^{2}\right)$, evolves according to

(8) $$dP=8\alpha_{z}^{2}{\left(1-P\right)}^{2}dt+4\alpha_{z}r_{z}\left(1-P\right)dW.$$

The dynamics take the purity asymptotically towards a fixed point at P=1, or the density matrix towards one of $\rho_{\pm }^{e}$, which is clearly a natural consequence of the process of measurement.

**Figure 3:**
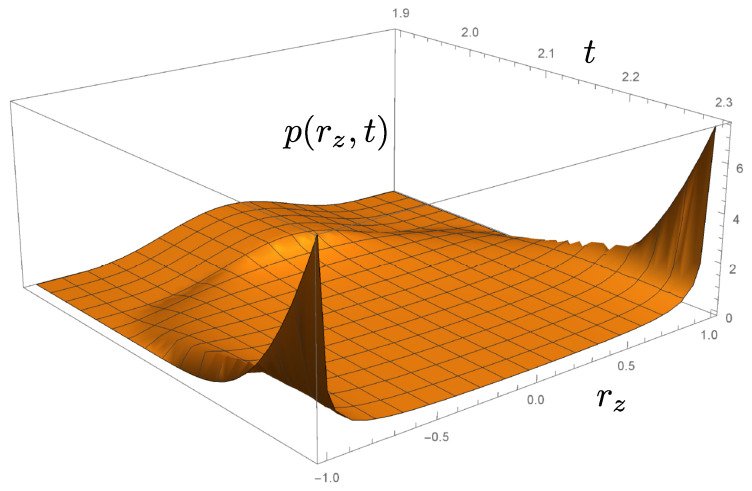
A probability density function $p(r_{z},t)$, evolving according to the Fokker–Planck Equation (9), describing the evolution of an ensemble of density matrices under measurement of $\sigma_{z}$. A Gaussian centred initially at the origin separates and probability density accumulates asymptotically at $r_{z}=\pm 1$. This approach complements the direct computation of trajectories $r_{z}\left(t\right)$ illustrated in Figure 2.

The Fokker–Planck equation describing the evolution of the probability density function (pdf) $p(r_{z},t)$ for the system variable $r_{z}$ is

(9) $$\frac{\partial p}{\partial t}=\frac{\partial^{2}}{\partial r_{z}^{2}}\left(2\alpha_{z}^{2}{\left(1-r_{z}^{2}\right)}^{2}p\right),$$

and this provides further insight into the dynamics. Figure 3 illustrates the development starting from a Gaussian pdf centred on the maximally mixed state at $r_{z}=0$. The ensemble of density matrices is separated by the dynamics into equal size groups that evolve asymptotically towards the eigenstates of $\sigma_{z}$ at $r_{z}=\pm 1$. The preservation of the ensemble average of $r_{z}$ is apparent.

### 2.2. Stochastic Entropy Production

The (total) stochastic entropy production associated with the evolution of a stochastic variable in a certain time interval is defined in terms of probabilities for the generation of a ‘forward’ set of moves in the phase space and the corresponding ‘backward’ set [4]. For the coordinate $r_{z}$ and the time interval dt, we need to consider the quantity

(10) $$\begin{array}{cc} \; & d\Delta s_{\mathrm{tot}}\left(r_{z},t\to r_{z}+dr_{z},t+dt\right)\\ \; & \;=\ln\left(\mathrm{Prob}(\mathrm{forward})/\mathrm{Prob}(\mathrm{backward})\right)\\ \; & \;=\ln\frac{p\left(r_{z},t\right)\Delta r_{z}\left(r_{z}\right)T\left(r_{z}\to r_{z}+dr_{z}\right)}{p\left(r_{z}+dr_{z},t+dt\right)\Delta r_{z}\left(r_{z}+dr_{z}\right)T\left(r_{z}+dr_{z}\to r_{z}\right)}, \end{array}$$

where the *T* are conditional probabilities for the transitions indicated. For stochastic variables that are odd under time reversal symmetry, additional features have to be included in this definition, but since $r_{z}$ is even, we can ignore such complications [7,48].

It may be shown that the expectation or mean of $d\Delta s_{\mathrm{tot}}$ is never negative, which ultimately provides an underpinning for the second law of thermodynamics [4].

We shall discuss the contributions to $d\Delta s_{\mathrm{tot}}$ involving the pdf $p(r_{z},t)$ and the volume increment $\Delta r_{z}\left(r_{z}\right)$ shortly, but first, let us consider the ratio of conditional probabilities. The two choices of forward move $\rho \to {\rho \prime }^{\pm }$ in Equations (1) and (2) are selected with probabilities

(11) $$p_{\pm }=\frac{1}{2}\left(1\pm 2\alpha_{z}r_{z}\sqrt{dt}\right).$$

The corresponding backward moves ${\rho \prime }^{\pm }\to \rho$ are described by the quantum maps

(12) $$\rho =\frac{{\tilde{M}}_{\mp }{\rho \prime }^{\pm }{\tilde{M}}_{\mp }^{†}}{\mathrm{Tr}\left({\tilde{M}}_{\mp }{\rho \prime }^{\pm }{\tilde{M}}_{\mp }^{†}\right)},$$

in terms of reverse Kraus operators ${\tilde{M}}_{\mp }$ that can be identified from the condition that the initial density matrix is recovered. Inserting Equation (1) into Equation (12), we have

(13) $$\rho =\frac{{\tilde{M}}_{\mp }M_{\pm }\rho M_{\pm }^{†}{\tilde{M}}_{\mp }^{†}}{\mathrm{Tr}\left({\tilde{M}}_{\mp }M_{\pm }\rho M_{\pm }^{†}{\tilde{M}}_{\mp }^{†}\right)},$$

which requires ${\tilde{M}}_{\mp }M_{\pm }$ to be proportional to the identity, up to linear order in dt. For $c=c^{†}$, this can be achieved using

(14) $${\tilde{M}}_{\mp }=\frac{1}{\sqrt{2}}\left(I-\frac{1}{2}c^{2}dt\mp c\sqrt{dt}\right)=M_{\mp },$$

and specifically for $c=\alpha_{z}\sigma_{z}$, we have

(15) $${\tilde{M}}_{\mp }M_{\pm }=\frac{1}{2}\left(1-2\alpha_{z}^{2}dt\right)I.$$

Hence, the probabilities for the backward moves are

(16) $$p_{\mp }^{\prime }=\mathrm{Tr}\left({\tilde{M}}_{\mp }{\rho \prime }^{\pm }{\tilde{M}}_{\mp }^{†}\right)=\frac{\mathrm{Tr}\left(M_{\mp }M_{\pm }\rho M_{\pm }^{†}M_{\mp }^{†}\right)}{\mathrm{Tr}\left(M_{\pm }\rho M_{\pm }^{†}\right)},$$

leading to

(17) $$p_{\mp }^{\prime }=\frac{\left(1-4\alpha_{z}^{2}dt\right)}{2\left(1\pm 2\alpha_{z}r_{z}\sqrt{dt}\right)}.$$

The ratio of conditional probabilities $T\left(r_{z}\to r_{z}+dr_{z}^{\pm }\right)/T\left(r_{z}+dr_{z}^{\pm }\to r_{z}\right)$ is then

(18) $$\frac{p_{\pm }}{p_{\mp }^{\prime }}=1\pm 4\alpha_{z}r_{z}\sqrt{dt}+4\alpha_{z}^{2}\left(1+r_{z}^{2}\right)dt.$$

**Figure 4:**
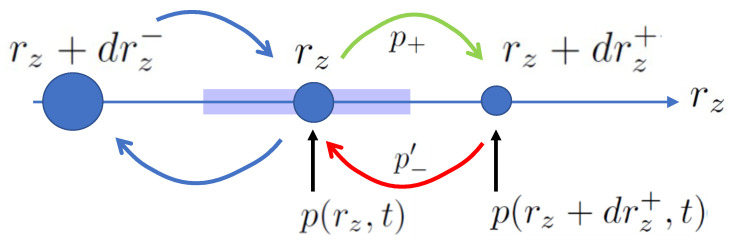
Available incremental moves on a set of locations on the $r_{z}$ axis according to the stochastic dynamics of measurement of $\sigma_{z}$, illustrating Equations (11), (17) and (19). The size of the circles represents the local probability density $p(r_{z},t)$. The shaded rectangle represents the volume $\Delta r_{z}=\frac{1}{2}\left(dr_{z}^{+}-dr_{z}^{-}\right)$ of the continuum phase space associated with a given location $r_{z}$.

The two possible increments in $r_{z}$ are

(19) $$\begin{array}{cc} dr_{z}^{\pm } & =\mathrm{Tr}\left({\rho \prime }^{\pm }\sigma_{z}\right)-r_{z}\\ \; & =-4\alpha_{z}^{2}r_{z}\left(1-r_{z}^{2}\right)dt\pm 2\alpha_{z}\left(1-r_{z}^{2}\right)\sqrt{dt}, \end{array}$$

and we note that the mean and variance over the two possibilities are

(20) $$\langle dr_{z}\rangle =p_{+}dr_{z}^{+}+p_{-}dr_{z}^{-}=0,$$

and

(21) $$\begin{array}{cc} \sigma_{r_{z}}^{2} & =p_{+}{\left(dr_{z}^{+}-\langle dr_{z}\rangle \right)}^{2}+p_{-}{\left(dr_{z}^{-}-\langle dr_{z}\rangle \right)}^{2}\\ \; & =4\alpha_{z}^{2}{\left(1-r_{z}^{2}\right)}^{2}dt, \end{array}$$

confirming that the evolution is consistent with the SDE for $r_{z}$ in Equation (7). The moves and their probabilities are illustrated in Figure 4.

We now write

(22) $$d\Delta s_{\mathrm{tot}}^{\pm }=d\Delta s_{A}^{\pm }+d\Delta s_{B}^{\pm },$$

where

(23) $$d\Delta s_{A}^{\pm }=\ln\left(\frac{T(r_{z}\to r_{z}+dr_{z}^{\pm })}{T(r_{z}+dr_{z}^{\pm }\to r_{z})}\right)=\ln\left(\frac{p_{\pm }}{p_{\mp }^{\prime }}\right),$$

and

(24) $$d\Delta s_{B}^{\pm }=\ln\left(\frac{p\left(r_{z},t\right)\Delta r_{z}\left(r_{z}\right)}{p\left(r_{z}+dr_{z}^{\pm },t+dt\right)\Delta r_{z}\left(r_{z}+dr_{z}^{\pm }\right)}\right).$$

Inserting Equation (18), we have

(25) $$\begin{array}{cc} \; & d\Delta s_{A}^{\pm }=\pm 4\alpha_{z}r_{z}\sqrt{dt}+4\alpha_{z}^{2}\left(1-r_{z}^{2}\right)dt, \end{array}$$

which provides two choices of incremental contribution to the stochastic entropy production in the forward move. We can compute the mean of $d\Delta s_{A}^{\pm }$:

(26) $$\begin{array}{cc} \langle d\Delta s_{A}\rangle & =p_{+}d\Delta s_{A}^{+}+p_{-}d\Delta s_{A}^{-}\\ \; & =\left(p_{+}-p_{-}\right)4\alpha_{z}r_{z}\sqrt{dt}+\left(p_{+}+p_{-}\right)4\alpha_{z}^{2}\left(1-r_{z}^{2}\right)dt\\ \; & =4\alpha_{z}^{2}\left(1+r_{z}^{2}\right)dt, \end{array}$$

and the variance:

(27) $$\begin{array}{cc} \sigma_{A}^{2} & =p_{+}{\left(d\Delta s_{A}^{+}-\langle d\Delta s_{A}\rangle \right)}^{2}+p_{-}{\left(d\Delta s_{A}^{-}-\langle d\Delta s_{A}\rangle \right)}^{2}\\ \; & =16\alpha_{z}^{2}r_{z}^{2}dt, \end{array}$$

from which we conclude that the evolution can be represented by an Itô process for a stochastic variable $\Delta s_{A}$:

(28) $$\begin{array}{cc} d\Delta s_{A} & =4\alpha_{z}^{2}\left(1+r_{z}^{2}\right)dt+4\alpha_{z}r_{z}dW. \end{array}$$

We next consider the contribution $d\Delta s_{B}^{\pm }$ to the stochastic entropy production given in Equation (24). The volume $\Delta r_{z}\left(r_{z}\right)$ is the region bounded by increments $\frac{1}{2}dr_{z}^{\pm }$ starting from $r_{z}$. It is the patch of phase space associated with coordinate $r_{z}$, as illustrated in Figure 4. We write $\Delta r_{z}=\frac{1}{2}\left(dr_{z}^{+}-dr_{z}^{-}\right)=2\alpha_{z}\left(1-r_{z}^{2}\right)\sqrt{dt}$ and then

(29) $$d\Delta s_{B}^{\pm }=-d\ln p^{\pm }+d\Delta s_{C}^{\pm },$$

where $d\ln p^{\pm }=\ln p\left(r_{z}+dr_{z}^{\pm },t+dt\right)-\ln p\left(r_{z},t\right)$ and

(30) $$\begin{array}{cc} d\Delta s_{C}^{\pm } & =\ln\left(\frac{\Delta r_{z}\left(r_{z}\right)}{\Delta r_{z}\left(r_{z}+dr_{z}^{\pm }\right)}\right)\\ \; & =4\alpha_{z}^{2}\left(1-r_{z}^{2}\right)dt\pm 4\alpha_{z}r_{z}\sqrt{dt}. \end{array}$$

The mean of $d\Delta s_{C}^{\pm }$ is

(31) $$\begin{array}{cc} \langle d\Delta s_{C}\rangle & =p_{+}d\Delta s_{C}^{+}+p_{-}d\Delta s_{C}^{-}\\ \; & =4\alpha_{z}^{2}\left(1+r_{z}^{2}\right)dt, \end{array}$$

and the variance is

(32) $$\begin{array}{cc} \sigma_{C}^{2} & =p_{+}{\left(d\Delta s_{C}^{+}-\langle d\Delta s_{C}\rangle \right)}^{2}+p_{-}{\left(d\Delta s_{C}^{-}-\langle d\Delta s_{C}\rangle \right)}^{2}\\ \; & =16\alpha_{z}^{2}r_{z}^{2}dt, \end{array}$$

so the Itô process for this component of stochastic entropy production is

(33) $$\begin{array}{cc} d\Delta s_{C} & =4\alpha_{z}^{2}\left(1+r_{z}^{2}\right)dt+4\alpha_{z}r_{z}dW. \end{array}$$

Similarly, it may be shown that the term $-d\ln p^{\pm }$ in Equation (29) makes a contribution of −dlnp to the Itô process for $d\Delta s_{\mathrm{tot}}$. Combining this with Equations (22), (28), (29) and (33), the stochastic entropy production can be shown to evolve according to the Itô process

(34) $$d\Delta s_{\mathrm{tot}}=-d\ln p\left(r_{z},t\right)+8\alpha_{z}^{2}\left(1+r_{z}^{2}\right)dt+8\alpha_{z}r_{z}dW.$$

The term $-d\ln p(r_{z},t)$ is usually referred to as the stochastic entropy production of the system, $d\Delta s_{\mathrm{sys}}$. The remaining terms are then regarded as stochastic entropy production in the environment (in this case the measuring device), and denoted $d\Delta s_{\mathrm{env}}$ or $d\Delta s_{\mathrm{meas}}.$ Note that the evolution of the stochastic entropy production in Equation (34), with a system contribution that depends on the pdf $p(r_{z},t)$ over the phase space of the density matrix, is continuous. This is in contrast to implementations of stochastic entropy production in quantum mechanics that involve the probability distribution over eigenstates of the measured operator in the formalism, or that invoke projective measurements causing discontinuities that are potentially infinite in magnitude [37].

### 2.3. Derivation of $d\Delta s_{\mathrm{tot}}$ from the Dynamics

The derivation of $d\Delta s_{\mathrm{tot}}$ in the previous section is intricate, but there is an alternative approach that is much more straightforward [6,7] and does not require the identification of reverse Kraus operators [49]. Let us consider an Itô process for a stochastic variable *x* in the form

(35) $$dx=\left(A^{\mathrm{rev}}\left(x,t\right)+A^{\mathrm{irr}}\left(x,t\right)\right)dt+B\left(x,t\right)dW,$$

where the terms proportional to $A^{\mathrm{rev}}$ and $A^{\mathrm{irr}}$ represent modes of deterministic dynamics that satisfy and violate time reversal symmetry, respectively. Then, the stochastic entropy production is given by

(36) $$\begin{array}{ccc} d\Delta s_{\mathrm{tot}} & = & -d\ln p\left(x,t\right)+\frac{A^{\mathrm{irr}}}{D}dx-\frac{A^{\mathrm{rev}}A^{\mathrm{irr}}}{D}dt+\frac{\partial A^{\mathrm{irr}}}{\partial x}dt\\ & & -\frac{\partial A^{\mathrm{rev}}}{\partial x}dt-\frac{1}{D}\frac{\partial D}{\partial x}dx+\frac{\left(A^{\mathrm{rev}}-A^{\mathrm{irr}}\right)}{D}\frac{\partial D}{\partial x}dt\\ & & -\frac{\partial^{2}D}{\partial x^{2}}dt+\frac{1}{D}{\left(\frac{\partial D}{\partial x}\right)}^{2}dt, \end{array}$$

where $D\left(x,t\right)=\frac{1}{2}B{\left(x,t\right)}^{2}$. This expression might not seem particularly intuitive, but for dynamics that possess a stationary state with zero probability current, characterised by a pdf $p_{\mathrm{st}}\left(x\right)$, Equation (36) reduces to the simpler expression $d\Delta s_{\mathrm{tot}}=-d\ln\left(p\left(x,t\right)/p_{\mathrm{st}}\left(x\right)\right)$, and hence, the stochastic entropy production is seen to arise from deviation from stationarity.

For the dynamics of $r_{z}$ given by Equation (7), we have $A^{\mathrm{rev}}=A^{\mathrm{irr}}=0$ and $B=2\alpha_{z}\left(1-r_{z}^{2}\right)$. Hence, $D=2\alpha_{z}^{2}{\left(1-r_{z}^{2}\right)}^{2}$, leading to $dD/dr_{z}=-8\alpha_{z}^{2}r_{z}\left(1-r_{z}^{2}\right)$, $d^{2}D/dr_{z}^{2}=-8\alpha_{z}^{2}\left(1-3r_{z}^{2}\right)$, and

(37) $$\begin{array}{cc} d\Delta s_{\mathrm{tot}} & =-d\ln p-\frac{1}{D}\frac{dD}{dr_{z}}dr_{z}-\frac{d^{2}D}{dr_{z}^{2}}dt+\frac{1}{D}{\left(\frac{dD}{dr_{z}}\right)}^{2}dt\\ \; & =-d\ln p+8\alpha_{z}^{2}\left(1+r_{z}^{2}\right)dt+8\alpha_{z}r_{z}dW. \end{array}$$

This is in agreement with Equation (34), but the derivation is much more direct. Extension to sets of coupled Itô processes for several stochastic variables $\left\{x_{i}\right\}$ is straightforward, and we shall encounter an example of such a generalisation in Section 3.

### 2.4. Results

**Figure 5:**
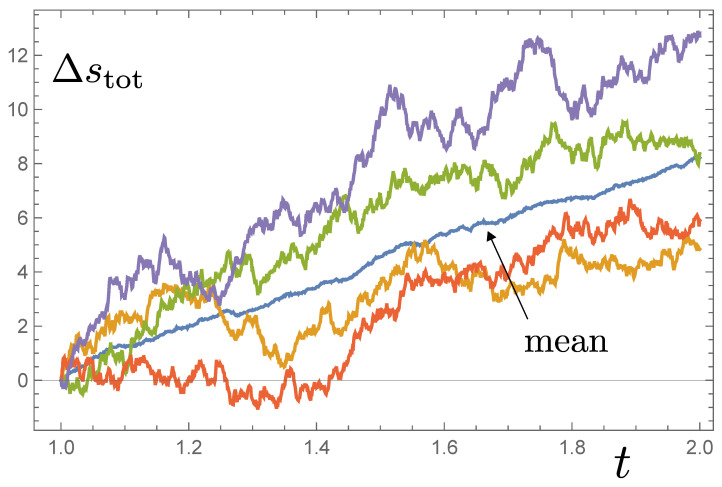
Four trajectories illustrating the stochastic entropy production $\Delta s_{\mathrm{tot}}\left(t\right)$ for the dynamics of Equation (7) in the interval 1≤t≤2, starting from a Gaussian pdf centred on $r_{z}=0$ at t=0, and with $\alpha_{z}=1$. The mean over a sample of 40 trajectories is consistent with an asymptotic average rate of production equal to $8\alpha_{z}^{2}$, as suggested in Equation (47).

Let us now consider the character of the stochastic entropy production described by Equation (37). It is straightforward to evaluate $\Delta s_{\mathrm{tot}}\left(t\right)$ numerically, employing solutions to the Fokker–Planck Equation (9) and the Itô process for $r_{z}\left(t\right)$. Example evolutions of $\Delta s_{\mathrm{tot}}\left(t\right)$ associated with trajectories $r_{z}\left(t\right)$ are shown in Figure 5, for $\alpha_{z}=1$. The mean stochastic entropy production over a sample of trajectories appears to rise linearly in time. The increase reflects the fact that the pdf $p(r_{z},t)$ does not reach a stationary state, but instead progressively sharpens towards two δ-function peaks at $r_{z}=\pm 1$. The system approaches one of the eigenstates but does not reach it in finite time. A system that continues to evolve in response to time reversal asymmetric dynamics (which includes the noise term as well as the deterministic contribution proportional to $A^{\mathrm{irr}}$ in Equation (35)) is characterised by stochastic entropy production.

The calculations of $\Delta s_{\mathrm{tot}}$ in Figure 5 were obtained after performing a transformation of the stochastic variable to avoid difficulties arising from the singularities in $p(r_{z},t)$ as t→∞. It is possible to do this since the stochastic entropy production is invariant under a coordinate transformation. Consider, then, the variable $y=\tanh^{-1}r_{z}$, which evolves in time according to

(38) $$dy=4\alpha_{z}^{2}\tanh y\;dt+2\alpha_{z}dW,$$

using Itô’s lemma. The phase space $-1\leq r_{z}\leq 1$ maps to −∞≤y≤∞. We identify $A^{\mathrm{rev}}\left(y\right)=0$, $A^{\mathrm{irr}}\left(y\right)=4\alpha_{z}^{2}\tanh y$, $D\left(y\right)=2\alpha_{z}^{2}$ and write

(39) $$\begin{array}{cc} \; & d\Delta s_{\mathrm{tot}}=-d\ln p\left(y,t\right)+\frac{A^{\mathrm{irr}}}{D}dy+\frac{dA^{\mathrm{irr}}}{dy}dt\\ \; & =-d\ln p\left(y,t\right)+4\alpha_{z}^{2}\left(1+\tanh^{2}y\right)dt+4\alpha_{z}\tanh y\;dW, \end{array}$$

where the pdf for *y* satisfies the Fokker–Planck equation

(40) $$\frac{\partial p}{\partial t}=-4\alpha_{z}^{2}\frac{\partial }{\partial y}\left(\tanh y\;p\right)+2\alpha_{z}^{2}\frac{\partial^{2}p}{\partial y^{2}}.$$

Solving Equations (38)–(40) numerically produces the trajectories in Figure 5.

We can perform an analysis of the evolution at late times, where $r_{z}$ is close to 1 or −1 such that |y| is large. The dynamics are then approximated by

(41) $$dy=\pm 4\alpha_{z}^{2}dt+2\alpha_{z}dW,$$

employing the plus sign if y>0 and the negative if y<0. The Fokker–Planck equation is

(42) $$\frac{\partial p}{\partial t}=-4\alpha_{z}^{2}\mathrm{sgn}\left(y\right)\frac{\partial p}{\partial y}+2\alpha_{z}^{2}\frac{\partial^{2}p}{\partial y^{2}},$$

which has an approximate asymptotic solution:

(43) $$p\left(y,t\right)\;\propto \;\frac{1}{t^{1/2}}\;\left[\exp\left[-\frac{{\left(y-4\alpha_{z}^{2}t\right)}^{2}}{8\alpha_{z}^{2}t}\right]+\exp\left[-\frac{{\left(y+4\alpha_{z}^{2}t\right)}^{2}}{8\alpha_{z}^{2}t}\right]\right],$$

consisting of two Gaussians in the *y* phase space, drifting with equal and opposite velocities towards ±∞ and simultaneously broadening.

From Equation (39), we obtain stochastic entropy production for a trajectory with y≫0 of

(44) $$d\Delta s_{\mathrm{tot}}\approx -d\ln p_{+}\left(y,t\right)+8\alpha_{z}^{2}dt+4\alpha_{z}\;dW,$$

with

(45) $$p_{+}\propto \frac{1}{t^{1/2}}\exp\left(-\frac{{\left(y-4\alpha_{z}^{2}t\right)}^{2}}{8\alpha_{z}^{2}t}\right),$$

and hence,

(46) $$d\Delta s_{\mathrm{tot}}\approx d\left(\frac{{\left(y-4\alpha_{z}^{2}t\right)}^{2}}{8\alpha_{z}^{2}t}\right)+\frac{1}{2}d\ln t+8\alpha_{z}^{2}dt+4\alpha_{z}\;dW,$$

the average of which is

(47) $$\begin{array}{cc} d\langle \Delta s_{\mathrm{tot}}\rangle & \approx \frac{1}{t}dt-\frac{\langle {\left(y-4\alpha_{z}^{2}t\right)}^{2}\rangle }{8\alpha_{z}^{2}t^{2}}dt+8\alpha_{z}^{2}dt\\ \; & =\frac{1}{t}dt-\frac{4\alpha^{2}t}{8\alpha^{2}t^{2}}dt+8\alpha_{z}^{2}dt, \end{array}$$

which reduces to $8\alpha_{z}^{2}dt$ as t→∞. A similar conclusion can be reached if y≪0, so we expect mean stochastic entropy production at a constant rate $8\alpha_{z}^{2}$ as t→∞, confirming the behaviour seen in Figure 5.

### 2.5. Contrast with Von Neumann Entropy

At this point, we should consider whether stochastic entropy production is related to a change in the von Neumann entropy $S_{\mathrm{vN}}=-\mathrm{Tr}\rho \ln\rho$, a commonly employed expression for entropy in quantum mechanics.

The mean stochastic entropy production is the change in subjective uncertainty with regard to the quantum state adopted by the world. We are unable to make exact predictions when the dynamical influence of the environment on the system is not specified in detail. The dynamics then become effectively stochastic and our knowledge of the adopted state is reduced with time.

In contrast, the von Neumann entropy is the uncertainty inherent to a quantum state with regard to the outcomes of projective measurement in a basis in which the density matrix is diagonal. It is a Shannon entropy $-\sum_{i}P_{i}\ln P_{i}$ where $P_{i}$ is the probability of projection into eigenstate *i* of the observable. For a two-level system, the number of such outcomes is two, and so the von Neumann entropy has an upper limit of ln2.

In contrast, the upper limit of the mean stochastic entropy production, representing the change in uncertainty in the adopted quantum state of the world, is infinite, since there is a continuum of possible states that could be taken. The continued mean production of stochastic entropy associated with measurement, discussed in previous sections, represents this progressively greater uncertainty.

Note also that the stochastic entropy production we have been considering has no connection with heat transfer or work. The two-level system under consideration does not possess a Hamiltonian *H* and the adoption of one or the other level as a result of measurement does not involve a change in system energy; specifically, TrHρ=0 throughout. Stochastic entropy production is not necessarily associated with the dissipation of potential energy into heat. Indeed, it need not be in classical mechanics, for example in the free expansion of an ideal gas. In both classical and quantum settings, the purpose of entropy is to specify the degree of configurational uncertainty of a system. In classical mechanics, the configurations are described by sets of classical coordinates; in quantum mechanics, they are specified by collections of (reduced) density matrix elements.

**Figure 6:**
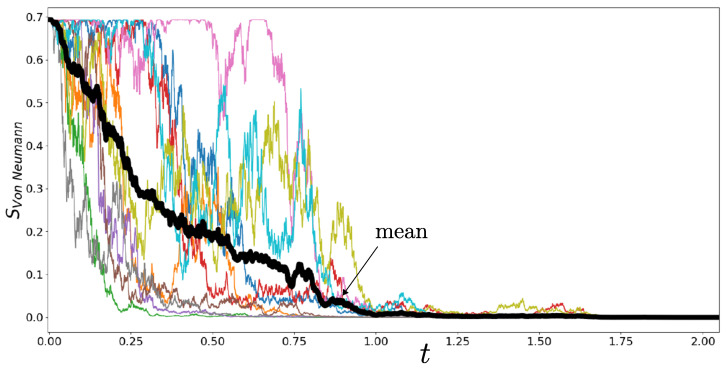
Evolution of the von Neumann entropy of the reduced density matrix of the two-level system, for 10 stochastic trajectories governed by the dynamics of Equation (7) with $\alpha_{z}=1$. Mean behaviour is also shown. Asymptotic values of zero imply that the system is purified.

Von Neumann entropy does play a role in computing the thermodynamic entropy of a quantum system in a situation where it is subjected to projective measurement and thereafter regarded as occupying one of the eigenstates. However, it is not straightforward to employ von Neumann entropy in discussions of the second law and the arrow of time. The first issue is that the von Neumann entropy $-\mathrm{Tr}\bar{\rho }\ln\bar{\rho }$ of the ensemble averaged density matrix $\bar{\rho }$ remains constant under the measurement dynamics employed here (because $\bar{\rho }$ remains constant). In contrast, the von Neumann entropy of a typical member of the considered ensemble of density matrices falls to zero under the dynamics. This is illustrated in Figure 6 for the two-level system where ρ evolves towards one of the $\rho_{\pm }^{e}$; the latter are pure states with $S_{\mathrm{vN}}=0$. The mean von Neumann entropy change −ΔTr〈ρlnρ〉 associated with the measurement process is then negative. In order to protect the second law, we need to consider entropy change in the environment. The total stochastic entropy production includes such a contribution and so provides a more inclusive framework for discussions of irreversibility.

## 3. Simultaneous Measurement of $\sigma_{z}$ and $\sigma_{x}$

### 3.1. Evolution Towards Purity

Now we turn our attention to a more complicated case of stochastic entropy production associated with the dynamics of an open quantum system. We continue to use the framework of quantum state diffusion, involving transformations according to Equation (1), but we now represent the stochastic influence of the environment on the system using *two* pairs of Kraus operators, given by

(48) $$\begin{array}{cc} M_{1\pm } & =\frac{1}{2}\left(I-\frac{1}{2}c_{1}^{†}c_{1}dt\pm c_{1}\sqrt{dt}\right)\\ M_{2\pm } & =\frac{1}{2}\left(I-\frac{1}{2}c_{2}^{†}c_{2}dt\pm c_{2}\sqrt{dt}\right), \end{array}$$

with $c_{1}=\alpha_{z}\sigma_{z}$ and $c_{2}=\alpha_{x}\sigma_{x}$. The first and second pairs describe the dynamics of continuous measurement of observables $\sigma_{z}$ and $\sigma_{x}$, respectively, and together therefore represent the performance of simultaneous measurement. Since $\sigma_{z}$ and $\sigma_{x}$ do not commute, we expect this not to result in a fixed outcome, and quantum state diffusion provides an interesting illustration of what this means.

**Figure 7:**
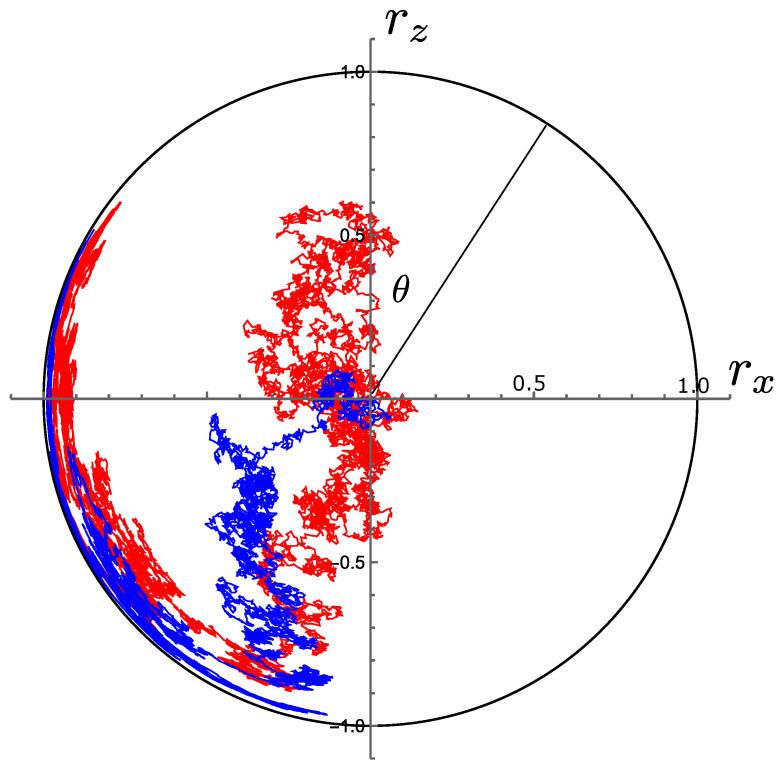
Two trajectories of the density matrix coordinates ($r_{x}\left(t\right),r_{z}\left(t\right)$) generated by the dynamics of simultaneous measurement of $\sigma_{x}$ and $\sigma_{z}$, Equation (50), starting from the maximally mixed state at the origin and for equal strengths of measurement $\alpha_{x}$ and $\alpha_{z}$. The outer black circle represents a condition of purity, towards which the system evolves. Eigenstates of $\sigma_{x}$ and $\sigma_{z}$ lie at θ=±π/2 and θ=0,π on the circle, respectively.

**Figure 8:**
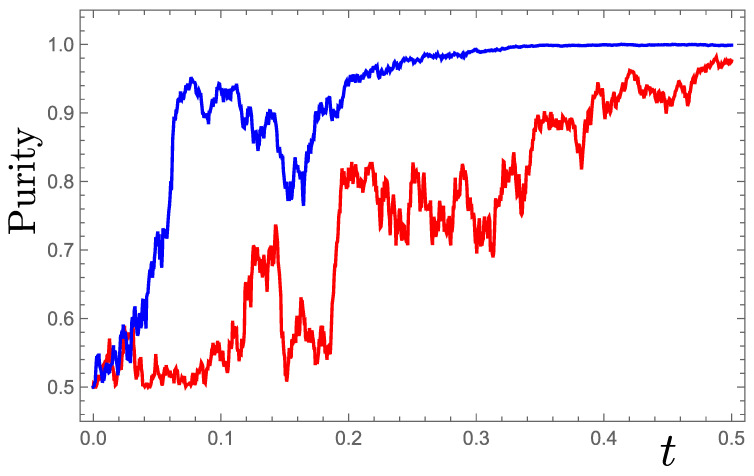
Evolution of purity for the system trajectories in Figure 7.

Probabilities of stochastic changes in the reduced density matrix of the system, brought about by interactions with the environment, may be deduced for these operators, and a stochastic Lindblad equation for its evolution may be derived:

(49) $$\begin{array}{cc} d\rho & =\sum_{i=1,2}\left(c_{i}\rho c_{i}^{†}-\frac{1}{2}\rho c_{i}^{†}c_{i}-\frac{1}{2}c_{i}^{†}c_{i}\rho \right)dt\\ \; & \;+\left(\rho c_{i}^{†}+c_{i}\rho -C_{i}\rho \right)dW_{i}, \end{array}$$

with $C_{i}=\mathrm{Tr}\left((c_{i}+c_{i}^{†})\rho \right)$. Upon inserting the representation $\rho =\frac{1}{2}\left(I+r_{z}\sigma_{z}+r_{x}\sigma_{x}\right)$, the dynamics can be expressed as

(50) $$\begin{array}{cc} dr_{z} & =2\alpha_{z}\left(1-r_{z}^{2}\right)dW_{z}-2\alpha_{x}^{2}r_{z}dt-2\alpha_{x}r_{z}r_{x}dW_{x}\\ dr_{x} & =2\alpha_{x}\left(1-r_{x}^{2}\right)dW_{x}-2\alpha_{z}^{2}r_{x}dt-2\alpha_{z}r_{x}r_{z}dW_{z}, \end{array}$$

where $dW_{x}$ and $dW_{z}$ are independent Wiener increments. Example stochastic trajectories starting from the maximally mixed state at $r_{x}=r_{z}=0$ are shown in Figure 7. The purity $P=\mathrm{Tr}\rho^{2}=\frac{1}{2}\left(1+r^{2}\right)$, where $r^{2}=r_{x}^{2}+r_{z}^{2}$, evolves according to

(51) $$\begin{array}{cc} dP & =4\left(\alpha_{x}^{2}\left(1-r_{x}^{2}\right)+\alpha_{z}^{2}\left(1-r_{z}^{2}\right)\right)\left(1-P\right)dt\\ \; & +4\alpha_{x}r_{x}\left(1-P\right)dW_{x}+4\alpha_{z}r_{z}\left(1-P\right)dW_{z}, \end{array}$$

such that P=1 is a fixed point reached asymptotically in time. Examples of such system purification are shown in Figure 8.

The dynamics can be recast in terms of $Y=\tanh^{-1}r^{2}$, which tends to *∞* as r→1, and an angle $\theta =\tan^{-1}\left(r_{x}/r_{z}\right)$. For $\alpha_{x}=\alpha_{z}=\alpha$, the SDEs for these variables are

(52) $$\begin{array}{cc} dY & =\frac{4\alpha^{2}}{{\left(1+\tanh Y\right)}^{2}}\left(2+\tanh Y+3\tanh^{2}Y\right)dt\\ \; & \;+\frac{4\alpha \sqrt{\tanh Y}}{1+\tanh Y}dW_{Y}\\ d\theta & =2\alpha \;dW_{\theta }/\sqrt{\tanh Y}, \end{array}$$

where $dW_{Y}=r^{-1}\left(r_{z}dW_{z}+r_{x}dW_{x}\right)$ and $dW_{\theta }=r^{-1}\left(-r_{x}dW_{z}+r_{z}dW_{x}\right)$ are independent Wiener increments. As t→∞, Equation (51) implies that $r^{2}\to 1$ and hence tanhY→1, in which case we can write

(53) $$dY\approx 6\alpha^{2}dt+2\alpha \;dW_{Y},$$

and so for late times, we have $Y\approx 6\alpha^{2}t+2\alpha W_{Y}+\mathrm{const}$. The SDE for θ in this limit is $d\theta =2\alpha dW_{\theta }$, such that the pdf becomes uniform over θ. We write $p\left(Y,\theta ,t\right)\to {\left(2\pi \right)}^{-1}F\left(Y,t\right)$, in terms of a travelling and broadening Gaussian in *Y*:

(54) $$F\left(Y,t\right)=\frac{1}{{\left(8\pi \alpha^{2}t\right)}^{1/2}}\exp\left[-\frac{{\left(Y-6\alpha^{2}t\right)}^{2}}{8\alpha^{2}t}\right].$$

The stochastic entropy production can now be computed using the framework of *Y* and θ coordinates. We shall do so first for late times where Y→1 and the dynamical Equation (52) become independent. We can identify coefficients $A_{Y}^{\mathrm{irr}}=6\alpha^{2}$, $A_{Y}^{\mathrm{rev}}=0$, $D_{Y}=2\alpha^{2}$, and $A_{\theta }^{\mathrm{irr}}=0$, $A_{\theta }^{\mathrm{rev}}=0$, $D_{\theta }=2\alpha^{2}$ and use Equation (36) to identify contributions to the stochastic entropy production. The system stochastic entropy production can be computed using the pdf in Equation (54). After some manipulation, we find that

(55) $$d\Delta s_{\mathrm{tot}}\approx 18\alpha^{2}dt+6\alpha \;dW_{Y},$$

and thus, the stochastic entropy production increases at a mean rate of $18\alpha^{2}$. This is more than twice the mean rate of production in Equation (46) for the case of measurement of $\sigma_{z}$ alone. The continued increase is once again a consequence of the non-stationary character of the evolution; the dynamics have the effect of purifying the system, but only as t→∞.

**Figure 9:**
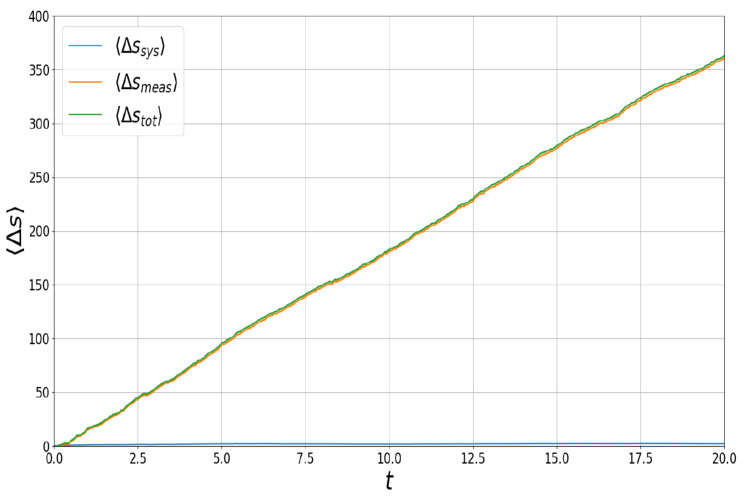
Mean stochastic entropy production $\langle \Delta s_{\mathrm{tot}}\rangle$ for simultaneous measurement of observables $\sigma_{x}$ and $\sigma_{z}$, separated into contributions associated with the system and measuring device, $\langle \Delta s_{\mathrm{sys}}\rangle$ and $\langle \Delta s_{\mathrm{meas}}\rangle$, respectively. The strengths of measurement $\alpha_{x}$ and $\alpha_{z}$ are both set to unity and the numerically generated ensemble is composed of ten trajectories. The mean stochastic entropy production is consistent with the estimate in Equation (55).

For the more general situation, without taking *t* to be large, it is possible to compute the stochastic entropy production numerically, based on the more elaborate coefficients of the SDEs in Equation (52), and a general solution to the associated Fokker–Planck equation. Mean stochastic entropy production over an ensemble of 10 trajectories is given in Figure 9, separating $\langle \Delta s_{\mathrm{tot}}\rangle$ into contributions $\langle \Delta s_{\mathrm{sys}}\rangle =-\Delta \langle \ln p\rangle$ and $\langle \Delta s_{\mathrm{meas}}\rangle =\langle \Delta s_{\mathrm{tot}}\rangle -\langle \Delta s_{\mathrm{sys}}\rangle$. The significance of this separation is that

(56) $$\begin{array}{cc} -\Delta \langle \ln p\rangle & =-\int p(Y,\theta ,t)\ln p(Y,\theta ,t)dYd\theta \\ \; & +\int p(Y,\theta ,0)\ln p(Y,\theta ,0)dYd\theta , \end{array}$$

is the change in Gibbs entropy $\Delta S_{G}$ of the system when described using the pdf in Y,θ coordinates. Note that the Gibbs entropy is coordinate frame-dependent and is therefore a measure of the uncertainty of adopted state in a specific coordinate system. In contrast, the mean stochastic entropy production is independent of coordinate frame.

### 3.2. Measurement of Two Non-Commuting Observables for a Pure State

Simultaneous measurement of $\sigma_{z}$ and $\sigma_{x}$ leads asymptotically to a pure state located on a circle of radius $r=\sqrt{r_{x}^{2}+r_{z}^{2}}=1$ in the $\left(r_{x},r_{z}\right)$ coordinate space. It is of interest now to consider how the pdf over the angle θ (shown in Figure 7) depends on the relative strengths of measurement of the two observables, and to compute the stochastic entropy production arising from changes in this ratio.

**Figure 10:**
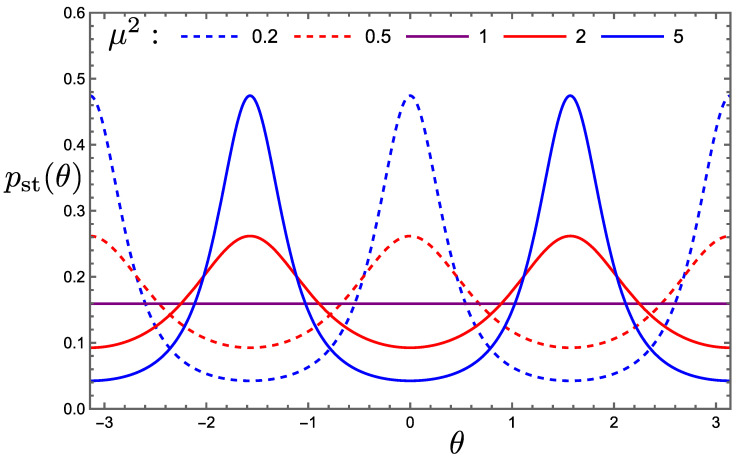
Stationary pdfs $p_{\mathrm{st}}\left(\theta \right)$ for simultaneous measurement of $\sigma_{x}$ and $\sigma_{z}$ with strengths $\alpha_{x}$ and $\alpha_{z}$, respectively, and strength ratio $\mu =\alpha_{x}/\alpha_{z}$, when the system is a pure state.

We therefore return to Equation (50), set $r_{x}=\sin\theta$, $r_{z}=\cos\theta$ and derive an SDE for θ in the form

(57) $$\begin{array}{cc} d\theta & =\left(\alpha_{x}^{2}-\alpha_{z}^{2}\right)\sin2\theta dt+2\alpha_{x}\cos\theta \;dW_{x}-2\alpha_{z}\sin\theta \;dW_{z}\\ \; & =\left(\alpha_{x}^{2}-\alpha_{z}^{2}\right)\sin2\theta dt+2{\left(\alpha_{x}^{2}\cos^{2}\theta +\alpha_{z}^{2}\sin^{2}\theta \right)}^{1/2}dW, \end{array}$$

which depends on the two measurement strengths $\alpha_{x}$ and $\alpha_{z}$, and where dW is a Wiener increment. The Fokker–Planck equation for the pdf p(θ,t) reads

(58) $$\begin{array}{cc} \frac{\partial p(\theta ,t)}{\partial t} & =-\frac{\partial }{\partial \theta }[\left(\alpha_{x}^{2}-\alpha_{z}^{2}\right)\sin2\theta \;p\left(\theta ,t\right) \end{array}$$

(59) $$\begin{array}{cc} \; & -2\frac{\partial }{\partial \theta }\left(\alpha_{x}^{2}\cos^{2}\theta +\alpha_{z}^{2}\sin^{2}\theta \right)p\left(\theta ,t\right)], \end{array}$$

and has stationary solutions (with zero probability current) given by

(60) $$p_{\mathrm{st}}\left(\theta \right)=\frac{\sqrt{2}\mu^{2}{\left(1+\mu^{2}-\left(1-\mu^{2}\right)\cos2\theta \right)}^{-3/2}}{E\left(1-\mu^{2}\right)+\mu E\left(1-\mu^{-2}\right)},$$

where $E\left(x\right)=\int_{0}^{\pi /2}{\left(1-x\sin^{2}\phi \right)}^{1/2}d\phi$ is the complete elliptical integral of the second kind and $\mu =\alpha_{x}/\alpha_{z}$ is the ratio of the two measurement strengths. Examples of stationary pdfs for various values of μ are shown in Figure 10. Clearly, a greater strength of measurement of observable $\sigma_{x}$ produces higher probability density in the vicinity of the eigenstates of $\sigma_{x}$ at θ=±π/2 than in the vicinity of the eigenstates of $\sigma_{z}$ at θ=0 and π, and vice versa.

Note that a form of Heisenberg uncertainty is exhibited by the stationary pdf. In quantum state diffusion, $r_{x}=\mathrm{Tr}\left(\rho \sigma_{x}\right)$ and $r_{z}=\mathrm{Tr}\left(\rho \sigma_{z}\right)$ are properties of the quantum state that are correlated in their evolution. The expectation value of each in the stationary state is zero:

(61) $$\begin{array}{cc} \langle r_{z}\rangle & =\int_{-\pi }^{\pi }\cos\theta \;p_{\mathrm{st}}\left(\theta \right)d\theta =0\\ \langle r_{x}\rangle & =\int_{-\pi }^{\pi }\sin\theta \;p_{\mathrm{st}}\left(\theta \right)d\theta =0, \end{array}$$

while the variances $\langle r_{z}^{2}\rangle -{\langle r_{z}\rangle }^{2}=\int_{-\pi }^{\pi }\cos^{2}\theta \;p_{\mathrm{st}}\left(\theta \right)d\theta$ and $\langle r_{x}^{2}\rangle -{\langle r_{x}\rangle }^{2}=\int_{-\pi }^{\pi }\sin^{2}\theta \;p_{\mathrm{st}}\left(\theta \right)d\theta$ sum to unity. A higher measurement strength for one observable drives up the variance of the associated variable (namely, the adopted values lie close to either 1 or −1) while driving down the variance of the other variable (the value of which lies close to zero).

The stochastic entropy production associated with the dynamics of θ is specified by $A_{\theta }^{\mathrm{rev}}=0$, $A_{\theta }^{\mathrm{irr}}=\left(\alpha_{x}^{2}-\alpha_{z}^{2}\right)\sin2\theta$, and $D_{\theta }=2\left(\alpha_{x}^{2}\cos^{2}\theta +\alpha_{z}^{2}\sin^{2}\theta \right)$, which leads to

(62) $$\begin{array}{cc} d\Delta s_{\mathrm{tot}} & =\left(6\left(\alpha_{x}^{2}-\alpha_{z}^{2}\right)\cos2\theta +\frac{9{\left(\alpha_{x}^{2}-\alpha_{z}^{2}\right)}^{2}\sin^{2}2\theta }{2\left(\alpha_{x}^{2}\cos^{2}\theta +\alpha_{z}^{2}\sin^{2}\theta \right)}\right)dt\\ \; & +\frac{3\left(\alpha_{x}^{2}-\alpha_{z}^{2}\right)\sin2\theta }{{\left(\alpha_{x}^{2}\cos^{2}\theta +\alpha_{z}^{2}\sin^{2}\theta \right)}^{1/2}}dW-d\ln p\left(\theta ,t\right). \end{array}$$

**Figure 11:**
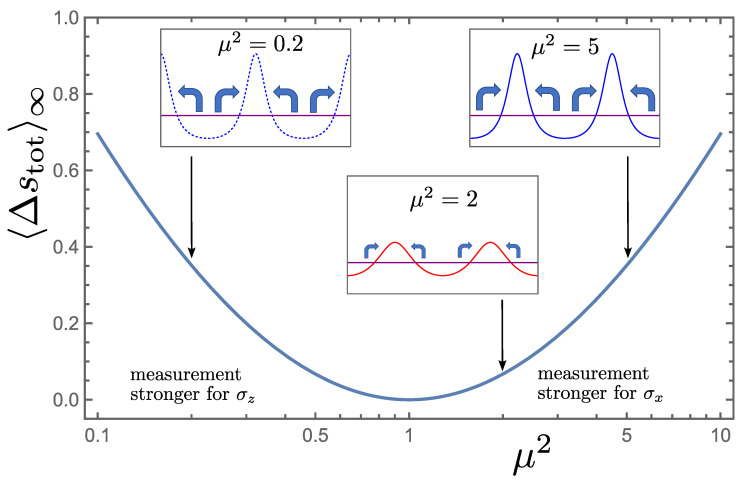
The asymptotic mean stochastic entropy production brought about by an abrupt change in the ratio $\mu =\alpha_{x}/\alpha_{z}$, starting from equal measurement strengths. The final stationary pdfs for $\mu^{2}=0.2$, 2 and 5, from Figure 10, as well as the initial uniform state, are shown in the insets together with arrows indicating the change in shape brought about by the process.

The dynamic and entropic consequences of changing the ratio of measurement strengths, for an initially pure state, can be established by solving Equations (57), (58) and (62) for a given protocol. However, we instead focus attention on a case with an analytic result. The asymptotic mean production of stochastic entropy for a transition from a uniform stationary pdf over θ, at equal measurement strengths $\alpha_{x}^{i}=\alpha_{z}^{i}$, to a final stationary state brought about by an abrupt change in measurement strengths to $\alpha_{x}^{f}=\mu \alpha_{z}^{f}$ at t=0, takes the form of a Kullback–Leibler divergence or relative entropy, an often used measure of distance between probability densities:

(63) $${\langle \Delta s_{\mathrm{tot}}\rangle }_{\infty }=\int p_{\mathrm{st}}^{i}\left(\theta \right)\ln\left(p_{\mathrm{st}}^{i}\left(\theta \right)/p_{\mathrm{st}}^{f}\left(\theta \right)\right)d\theta ,$$

where the $p_{\mathrm{st}}^{i,f}\left(\theta \right)$ correspond to Equation (60) with the insertion of $\alpha_{x}^{i,f}$ and $\alpha_{z}^{i,f}$. This can be derived by noting that $d\Delta s_{\mathrm{tot}}=-d\ln\left(p\left(\theta ,t\right)/p_{\mathrm{st}}\left(\theta \right)\right)$ in this case. We plot ${\langle \Delta s_{\mathrm{tot}}\rangle }_{\infty }$ for various ratios of final measurement strengths μ in Figure 11. Note that elevation of the measurement strength of one of the observables relative to the other leads to positive mean stochastic entropy production, in accordance with the second law, and the effect for enhanced measurement of $\sigma_{x}$ relative to $\sigma_{z}$ is the same as for enhanced measurement of $\sigma_{z}$, i.e., the same production emerges for ratios μ and 1/μ.

## 4. Interpretation

We return now to the physical interpretation of stochastic entropy production in open quantum systems. By analogy with situations in classical dynamics, the average of the stochastic entropy production $\Delta s_{\mathrm{tot}}$ that accompanies the evolution expresses change in subjective uncertainty concerning the details of the quantum state of the world. We have argued that this uncertainty is generated in the same way as in classical physics. We have taken the dynamical evolution of the world to be deterministic, but we do not or cannot attempt to solve the equations of motion for the coordinates exactly. We instead coarse-grain aspects of the description and employ a set of stochastic equations that capture the resulting unpredictability in evolution, again just as in a classical situation. Such modelling methods can only provide statistical predictions, and hence are characterised by an increase in entropy of (our perception of) the world. This is not a physical effect, but merely a measure of the absence of subjective knowledge, again just as in classical thermodynamics. The key point is that we take the quantum state vector of the world, and hence the reduced density matrix of an open system, to be the appropriate physical description, analogous to classical phase space coordinates.

It is possible to build such stochastic models from an underlying Hamiltonian describing the system and environment [42], but here we have adopted a more direct approach, using a framework of quantum state diffusion to represent the environmental disturbances. The resulting Markovian stochastic rules of evolution, specified by Kraus operators, are designed to drive a system continuously and (pseudo)randomly towards one of its eigenstates. This is our conception of the process of quantum measurement, in contrast to instantaneous projection. The resulting evolution of the reduced density matrix resembles a path taken by a Brownian particle, and it can be described using a Fokker–Planck equation for a pdf over a suitable phase space, or an Itô process that specifies a stochastic trajectory.

The purpose of stochastic entropy production, in both classical and quantum systems, is to provide a measure of the apparent irreversibility of evolution and hence an arrow of time. Both of these depend on the scale of the coarse-graining. The definition in Equation (10) involves a comparison between the likelihoods, computed according to the stochastic model employed, of forward and backward sequences of events. A departure of $\Delta s_{\mathrm{tot}}$ from zero indicates that the model dynamics generate one of these sequences preferentially; that the dynamics are irreversible in the sense of breaking time reversal symmetry. The preferred sequences will exhibit effects such as dispersion rather than assembly.

Nevertheless, parts of the world can become better defined as time evolves according to these models. Entropy production in a quantum framework can be used to characterise the approach of an open system towards an eigenstate under measurement, but also more generally towards a stationary state in some circumstances. Entropic cost of quantum measurement is analogous to such a cost in simple models of classical measurement [50]. Furthermore, we can conceive of quantum processes that are reversible, in the sense that the average of $\Delta s_{\mathrm{tot}}$ is zero. This would arise, as in classical circumstances, when the driving of the system, for example the rate of change of coupling to a measuring device, becomes quasistatic. Hence, quantum measurement need not be irreversible, neither in the dynamic nor in the entropic sense.

## 5. Conclusions

Entropy production represents increasing subjective uncertainty of microscopic configuration brought about by employing stochastic models of the dynamics instead of the underlying deterministic equations of motion that are responsible for complex, dispersive behaviour. These ideas can apply to quantum systems, for which we regard the reduced density matrix as a physical property analogous to a set of physical coordinates of a classical system. The reduced density matrix evolves pseudorandomly through interactions with an underspecified environment, which we represent in a minimal fashion using Kraus operators and a framework of Markovian quantum state diffusion. We concern ourselves with the uncertainty in the reduced density matrix that is actually adopted by the system. Stochastic entropy production can then be computed using analysis of the relative probabilities of forward and backward Brownian trajectories of the reduced density matrix.

The crucial features of quantum mechanics are captured by such a dynamics, in particular the stochastic selection of an eigenstate according to the Born rule. A further feature has been explored, for a simple two-level system, where the simultaneous measurement of two observables represented by non-commuting operators can be considered. The system is prevented from selecting an eigenstate of either operator, in line with expectation, and instead adopts a state of correlated stationary uncertainty with respect to the two observables.

The model of measurement used here has the effect of purifying the system, i.e., eliminating any initial entanglement between the system and its environment. The effect is a consequence of the simplicity of the model, but it is perfectly in line with the idea that a system takes an eigenstate of a system observable after the process of measurement. The final state of the environment (the measuring device) is nevertheless correlated with the final state of the system, and this is the means by which it is able to convey information about the system observable and preserve a record of the measurement.

We suggest that the reduced density matrix typically used to describe an open quantum system is an average over an ensemble of adoptable states, pure as well as those entangled with the environment. Moreover, the ensemble average is not suitable for modelling eigenstate selection, which takes place at the level of ensemble members. This problem is traditionally accommodated by introducing a process of projective measurement that takes place outside the regular dynamics and changes the ensemble average, but such a difficulty is not present when considering the dynamics of ensemble members.

The dynamics we employ therefore conceptualise quantum mechanics as the evolution of physical properties that behave in a complex but relatively unmysterious fashion. The quantum state is more than a provider of information about probabilities of projective measurement outcomes. The reduced density matrix, and by implication the quantum state vector of the world, are treated as physical coordinates and not merely bearers of information.

Using such a dynamical framework, the main purpose of this paper has been to provide explicit examples of stochastic entropy production for a simple open quantum system, and to suggest that this quantity is the most appropriate extension into the quantum regime of the modern concept of entropy production. We have studied stochastic entropy production for scenarios involving the measurement of one and then two observables. Mean stochastic entropy production in this context measures the change in subjective uncertainty concerning the adopted quantum state of the world. It never decreases, thus satisfying the second law of thermodynamics. The von Neumann entropy is a measure of uncertainty in measurement outcome, but compared to mean stochastic entropy production, it plays a rather different role. The connections between the two are worth exploring further.

## Data Availability

The original contributions presented in the study are included in the article, further inquiries can be directed to the corresponding author.
